# Retraction Note: Glyoxalase I inhibition induces apoptosis in irradiated MCF-7 cells via a novel mechanism involving Hsp27, p53 and NF-*κ*B

**DOI:** 10.1038/s41416-025-03042-0

**Published:** 2025-04-30

**Authors:** C Antognelli, I Palumbo, C Aristei, V N Talesa

**Affiliations:** 1 Department of Experimental Medicine, University of Perugia, Sant’Andrea delle Fratte, 06132 Perugia, Italy; 2 Radiation Oncology Section, University of Perugia, Sant’Andrea delle Fratte, 06132 Perugia, Italy

Retraction to: *British Journal of Cancer* 10.1038/bjc.2014.280, published online 10 June 2014

The Editor-in-Chief has retracted this article. After publication, concerns were raised regarding similarities in the western blot images presented in the Figures. Specifically:

- The western blots in Figs. 2b, 2d and 4e appear to contain duplicate bands.
- The cytosol β-actin and Bcl-XL blots in Fig. 5a appear highly similar to cytosol β-actin and Cyt *c* (rotated 180 degrees) in Fig. 5b (left).
- In Fig. 5b (right) and c, the mitochondria β-actin bands appear highly similar.
- The blots presented in Fig. 6d (left) appear highly similar to those in Fig. 6e (left).

The authors have stated that the blots are similar, but not identical. However, they have been unable to provide the raw data to support this statement. Due to the high number of potential issues, the Editor-in-Chief no longer has confidence in the presented data.

V N Talesa has stated on behalf of all authors that they disagree with this retraction.

